# Paediatric palliative approach in the era of disease modifying therapies in spinal muscular atrophy — a comprehensive landscape of French specialist physicians’ perspectives

**DOI:** 10.1186/s13023-026-04481-5

**Published:** 2026-08-05

**Authors:** Marie-Liesse Guenée, Claire Pilet, Hélène Martinez, Véronique Christophe, Matthias Schell

**Affiliations:** 1 Equipe de Recherche ESPPéRA, Centre Léon Bérard (CLB), Lyon, France; 2 Centre Hospitalier Universitaire d’Angers, Angers, France; 3 Département de Sciences Humaines et Sociales, Centre Léon Bérard (CLB), Lyon, France; 4 Centre de Recherche en Cancérologie de Lyon (CRCL), UMR Inserm, Université Claude Bernard Lyon 1 (UCBL), Lyon, 1052 - CNRS 5286 France

**Keywords:** Spinal muscular atrophy, Disease-modifying therapies, Palliative care, Neurologists, Physical Medicine and Rehabilitation, Paediatrics

## Abstract

**Background:**

The introduction of disease-modifying therapies (DMTs) has transformed the prognosis of children with spinal muscular atrophy (SMA), creating new uncertainties in defining paediatric palliative care (PPC). This study explores the perspectives of paediatric neurologists and paediatric physical medicine and rehabilitation (PM&R) specialists in France on the definition of a palliative care (PC) situation, the PC practices in SMA and the integration of PPC teams in this evolving context.

**Methods:**

We conducted a qualitative study with 16 French paediatric neurologists and paediatric PM&R specialists. Semi-structured interviews were thematically analysed and supported by reflexive practices and triangulation.

**Results:**

Five main categories emerged from the analysis, revealing the heterogeneous definition of a PC situation and of the widespread involvement of PPC teams in untreated children. In the case of the treated SMA type 1 children, the PPC teams were mainly not involved, although the clinical complexity and uncertainty were acknowledged, and many of these teams’ qualifications were recognised. This was hindered by several factors, including specialists’ self-perceived expertise, difficulties in distinguishing specific skills from those of other teams, and the challenge of introducing PC to parents. Facilitators included renaming PPC teams, ensuring availability outside office hours, and perceiving PPC professionals as colleagues.

**Conclusions:**

This study highlights the widespread dimension of the PC dimension in SMA type 0 and 1 and the heterogeneous perceptions of PPC in light of the prognostic uncertainty introduced by DMTs among specialists caring for SMA. Exploring the introduction of a PPC team as a family-centered choice in treated SMA type 1 children could facilitate practitioners’ choice in referring to a PPC team.

**Supplementary Information:**

The online version contains supplementary material available at https://doi.org/10.1186/s13023-026-04481-5.

## Introduction

Since 2017, three disease-modifying treatments (DMTs) have changed the life expectancy of children with spinal muscular atrophy (SMA). SMA is a progressive neuromuscular disease that evolves, prior to these DMTs, into progressive proximal muscle weakness and atrophy and, in the most severe cases, paralysis and premature death [1]. SMA is caused by a mutation in the SMN1 gene and is one of the most common neuromuscular diseases, with an estimated point-prevalence of 1–2 per 100,000 persons [2]. The clinical phenotypes of SMAs are classified into 5 forms: type 0, with an in utero unset, to type 4, with an adult beginning [3, 4]. The most frequent type is type 1 [2], a severe type leading on average to death within the first year of age without treatment [5]. Over the last few years, these DMTs—one gene therapy (onasemnogene abeparvovec) and the antisense oligonucleotide (nusinersen) and the small-molecule (risdiplam), both acting as SMN2 splicing modifiers [6–8]—have improved overall survival and the ability to achieve motor milestones [9–11], particularly in SMA type 1. However, there is heterogeneity in treatment outcomes, depending on the number of SMN2 copies – and thus on the SMA type - and the introduction timeframe [10]. Many complications associated with this disease still exist and require ventilatory and nutritional support and spinal bracing [12]. Some studies also reported a decline in bulbar disorders [13, 14] and cognitive impairment [15, 16] in type 1 SMA children. In these severe cases, quality of life seems to be affected despite treatment [17, 18], and there is still much uncertainty regarding improvements in clinical outcomes [19]. The possibility of severe disability raises ethical questions about treatment indications, and in certain countries, including France, professionals are choosing not to treat patients who already have serious symptoms at diagnosis, including all type 0 SMA children [20, 21].

In this context of complexity [22], palliative care (PC) could be helpful, as it is defined in paediatrics as “patient- and family-centered care that optimises quality of life by anticipating, preventing, and treating suffering” for children with chronic, complex, and/or life-limiting illnesses/life-threatening illnesses and their families by the National Hospice and Palliative Care Organisation (NHPCO) [23, 24]. This pertinence is underscored by international palliative care standards as life-threatening illnesses carry a high risk of premature death but also the possibility of long-term survival [25].

The importance of PC was advocated by neurologists and Physical Medicine and Rehabilitation (PM&R) specialists in their field [26–28], including in SMA, before the emergence of DMTs [29]. For PC specialists, SMA type 1 was also considered one of the predominant primary clinical conditions in PC [30, 31]. Currently, in situations where no active treatment is provided or is discontinued, a PC approach – defined as PC principles that could be applied by “all healthcare providers dealing with a situation in which PPC is required” [25] - is still actively advocated [21]. However, to date, specialists’ recognition of a palliative situation in patients undergoing these DMTs remains uncertain, and only a few studies have suggested that PPC could still have a strong effect on children’s quality of life and disease progression [32, 33].

In addition, the neuropalliative care approach is seen as a necessary skill for adult neurologists and could be caried out only by neurologic care teams according to them [27] with the aim of limiting specialised PC interventions to highly complex cases in a consultative model [34–36]. In paediatrics, international standards have defined three levels of care and expertise of PPC programs: specialised PPC were defined as provided by “healthcare professionals specifically working in PPC in a dedicated setting” whereas generalised PPC concerned PC provided by “specialists of a given disease with training in PPC” [25]. Although PPC is becoming more widespread, specialised PPC is still recent and under development in several countries [37, 38]. In France, specialised PPC was deployed nationwide between 2008 and 2012 as regional PPC teams [39]. Nowadays, there are 25 regional specialised PPC teams and three specialised mobile intrahospital teams. Each French region has a specialised PPC team. One of their primary functions is to provide secondary-level support to healthcare teams requesting assistance and to promote the PPC approach. In this context, the role of PPC specialists according to paediatric neurologists and paediatric PM&R specialists in SMA type 0 and 1 has yet to be determined. The referral to these PPC specialists could likewise be affected by the context of uncertain prognosis [40], even though these new situations are complex, and more invasive supportive care seems to arise [20].

In this context of DMTs, our study explores what constitutes the palliative approach adopted by neurologists and paediatric PM&R specializing in SMA, and how is it reflected in the involvement of specialized PPC teams for children with SMA type 0 and type 1.

We carried out a prospective nationwide qualitative study based on interviews with paediatric neurologists and paediatric PM&R specialists in SMA to investigate their understanding of the role and integration of the PC approach within the overall care trajectory of children with SMA type 0 and 1.

## Methods

### Recruitment, inclusion criteria and sampling strategy

The participants were paediatric neurologists and paediatric PM&Rs specialising in the treatment and care of newborns and children with SMA. All eligible physicians were part of the Neuromuscular Commission of the French Paediatric Neurology Society (Société Française de Neuropédiatrie). They were recruited through electronic means, including email and videoconferencing, after an exhaustive sampling method. They could request additional details about the study by these means, if needed.

No sample size was calculated a priori. Recruitment ended when data saturation was reached, i.e., when investigators observed that no new theme emerged from the interviews [41]. The enrolment and interviews took place from February to May 2025.

### Study population and their SMA patients’ characteristics

Among the 57 specialists in the Commission, 16 (28%) participated in the study. In total, interviews were conducted with 16 different physicians working in 15 different hospitals across 14 regions in France. All participants, except two, were based in university hospitals and none of them were based in rural areas. Fifteen transcripts were analysed. One interview was not recorded because of equipment failure. The average interview length was 53 min (35 min to 114 min). There was no repeat interview. On one occasion, an interview was conducted partly in the presence of the interviewee’s colleague.

The characteristics of this sample are presented in Appendix 3. The sample included 11 women and 5 men. Nine participants were under 50 years old, and 7 were over 50 years old. Thirteen of the 15 physicians were paediatric neurologists. Medical experience ranged from less than 5 years to more than 20 years, with the majority having more than 15 years of experience (10/16, 62.5%). All but one participant was affiliated with either a coordinating site, an affiliated site, or an expert centre of the French National Rare Disease Network for Neuromuscular Disorders (FILNEMUS). Five specialists were based in a region where neonatal screening was implemented and, as a result, they could provide follow-up for SMA type 1 children who had received presymptomatic treatment.

Each physician reported the number of new patients seen over the past year, specifying how many children with SMA were treated or untreated with DMTs. These characteristics are summarized in Appendix 4. The number of new patients diagnosed and followed during this period ranged from 0 to 7.

### Terminology

In this study, ‘treated patients’ refers only to SMA type 1 patients treated by DMTs, whereas ‘untreated patients’ refers to SMA type 0 and type 1 patients not treated by DMTs, although these terms could generally include other SMA types. The decision to treat patients with SMA in France follows a multidisciplinary national consultation meeting, taking a case-by-case approach for each new diagnosis. All patients with SMA type 0 are currently untreated, in accordance with the current French national consensus. This distinction was not applied in the demographic questions, and physicians reported all SMA types when providing the numbers of treated and untreated patients.

The terms PC, end-of-life (EOL), life-threatening illness and life-limiting illness are used in the Results section as understood and expressed by the participants. No standardised definitions were provided prior to the interviews, as the aim was to explore physicians’ own perceptions and conceptualisations of these notions.

### Data

The data are reported in accordance with the Consolidated Criteria for Reporting Qualitative Research and the Standards for Reporting Qualitative Research [42].

Individual semi-structured interviews were conducted by one researcher (MG, MD), doing her master’s degree in palliative research. The participants received information regarding the study’s context and the interviewer’s professional background (paediatric neurology and palliative care degrees). A 12-question guide developed by a group composed of researchers in health psychology, PC and PC specialists was used. The interview guide was pilot-tested with a paediatric neurologist not specialised in SMA and was refined over the course of the study on the basis of field experience. The guide is available in appendix 1.

The interviews took place at the participants’ hospitals or through videoconferencing. The interviews were recorded on a tape recorder after authorisation from the participant. Field notes were made after the interviews to acknowledge and reflect on the researcher’s subjectivity.

Transcriptions were manually conducted by MG. Transcripts, were pseudonymized, and all the data were securely stored. Data processing complied with the French Data Protection Authority’s (CNIL) recommendations regarding confidentiality and participant rights.

The participants provided oral consent (i.e., oral declaration by participants of their consent to participate in the research, applied to research projects involving non patient subjects who do not require a consent form) for the use of their data, in accordance with ethical guidelines. An information sheet outlining their rights and the purpose of the study was provided prior to participation. The participants could withdraw their data from the analysis at any time. The transcripts were not returned to the participants for comments or for feedback. No participants dropped out of the study.

A reflexive thematic analysis was conducted independently by two researchers using the 6 phases of thematic analysis by Braun and Clarke [43]. Reflexive practices refer here to the continuous process through which researchers critically examine their own assumptions, positionings and the potential influence on the research. The data were triangulated for the first five interviews: two researchers independently analysed data, methods, and perspectives and regularly discussed it to increase validity. NVivo 15^®^ was used for coding. After the codes were grouped into subthemes, a group of researcher and PC specialists reviewed the subthemes, defined and named the themes and conceived a conceptual map of the themes, which is available in Appendix 2.

A reflexive diary was used to document initial assumptions and evolving reflections, promoting critical self-awareness and maintaining an open stance during interviews and data analysis.

### Ethical consideration

“Paediatric palliative approach in the era of disease modifying therapies in spinal muscular atrophy — A comprehensive landscape of French specialist physicians’” was conducted with nonpatients’ human participants. This study was performed in line with the principles of the Declaration of Helsinki and was approved by the independent Research Ethics Committee of the Léon Bérard Centre on the 18th of December 2024 with the treatment number R202-000-005.

## Analysis/results

### Categories

Five main categories emerged from the interview analysis. The participants addressed their vision of PC in SMA type 0 and 1, modifications caused by DMTs in their daily practices, particularly regarding the introduction of specialised PPC teams, and the questions arising from changes in prognosis and associated uncertainties. A summary of these themes with exemplar quotes is reported below.

### Self-perceived efficacy in palliative care

The participants described their understanding of PC within their daily practice. This distinction allows them to differentiate between the PC they feel competent to provide themselves - their self-perceived efficacy in this era - and the specialised expertise they may seek from specialised PPC teams.

All the physicians reported encountering PC situations in routine practice, whether in the context of SMA type 0 and 1, profound intellectual and multiple disabilities, neurodegenerative diseases, or other conditions; therefore, they perceived PC care as an integral part of both paediatric neurology and PM&R. These illnesses were viewed as reflecting the complexity of care inherent to PC situations. Other situations, such as epilepsy or metabolic diseases, were mentioned only one or two times.

Ultimately, we provide palliative care because we do paediatric neurology, obviously. (Doctor 8)

As they viewed PC as inherent to their specialties, few neurologists expressed that PC should be provided directly by themselves, without involving a specialised PPC team.

Finally, I consider palliative care to be medicine, and that our job as doctors is also to provide palliative care, so to speak, or to accompany the child until death, if they are expected to die. That’s why, for me, it is not necessarily something separate from what we do. At least for me, it’s not separate. And above all, it’s not… It doesn’t necessarily have to be a different team. (Doctor 13)

When I heard Americans talking about palliative care and all that stuff in the… The palliative care team, whatever, I thought to myself, well, we’re… Palliative care, we’re all palliative carers, so, um…(Doctor 15)

Indeed, doctors reported possessing PC skills that, in certain situations, reduced the need for involvement from specialised PPC teams. These included managing neonates with type 0 SMA, developing individualised PC plans, prescribing appropriate EOL treatments, and tailoring care to the needs of the child and their family.

As I was saying, we don’t ask for type 0 babies in the neonatal unit because… Perhaps wrongly! But in any case, we tell ourselves that we can do it. (Doctor 10)

A few physicians emphasised that these competencies were developed primarily through clinical experience.

We remember many situations involving children we have supported where we were truly affected by it, so to speak, and I can see that with my young colleagues – who are great, by the way – but I don’t have… Well, they have the same sensitivity to things, but… The experience of supporting a person and a family of a child who is going to die… It’s something you acquire, it’s truly an experience, and it’s not something you can learn very easily from articles or books. (Doctor 11)

Given the frequency of PC situations in their hospital departments, they also highlighted the clinical skills their teams had developed.

Yeah, I think we provide support… I don’t know. Maybe one child every three weeks, I would say. (Doctor 9)

Although they acknowledge that they have PC skills, physicians reported that providing support for children with untreated type 1 SMA was particularly challenging, as it involved prolonged follow-up, greater uncertainty, and more intense emotional responses from both families and care teams.

I have no hidden agenda like ‘we should have treated them,’ but… I don’t know if others have said this, yet I find that the longer it lasts, the more complicated it becomes. (Doctor 8)

So, ultimately, this kind of support is completely different from… Type 0 kind of support. Because parents go through many different stages; it’s not at all linear. There are questions, doubts, anger that build up over time… So, yes, it’s a longer and more difficult process. (Doctor 10)

They also noted emotional difficulties in managing cases where patients had limited understanding of their condition or when caring simultaneously for two children with similar diagnoses but differing trajectories—one receiving DMTs and the other receiving PC.

That is to say that when we support one, we can still at the same time diagnose another and prepare gene therapy for the other. […] I find that it is, uh yes, emotionally it is a bit hard. (Doctor 9)

### General perception of the paediatric palliative approach

The participants identified the PC dimension in their practice and articulated their own current definition of the PC approach and how they apply it to SMA 0 and 1. They reflected on how this definition has evolved with the widespread use of DMTs and expressed differing opinions regarding the PC situation in the context of treated SMA children. This theme provides a basis for understanding the other categories since recognition of a PC situation may or may not lead to it being treated as such.

Most physicians identified PPC primarily as a form of support for children and their families, without clearly defining its scope. When describing clinical situations necessitating this support, they mainly associated PPC with critical situations or conditions lacking curative treatment.

However, this is truly palliative care, support, with symptomatic treatment, comfort, etc. (Doctor 6)

Among critical situations, nine participants emphasised that PC is not limited to EOL care and may last several years. One physician explicitly distinguished between PC and EOL care, describing them as two distinct clinical stages, with EOL care representing a phase that follows the broader PC trajectory.

And after palliative care… When you leave palliative care, when it is no longer relevant, you move on to end-of-life care. So, end-of-life care follows a different set of procedures. (Doctor 5)

In light of this representation, all the doctors agreed that untreated SMA is a PC situation and needs a PC approach. As the EOL was close, these cases were clearly recognised as PC situations, which is consistent with their overall understanding of PC

I only do palliative care here. Apart from spinal muscular atrophy, which we treat, but otherwise I only do palliative care. (Doctor 1)

However, even among SMA children treated by DMTs, all participants recognised the complexity of symptomatic SMA type 1. The complexity described varied between multiple and severe disabilities—a lack of sitting posture, respiratory deficiencies requiring night-time ventilation, and scoliosis—and variable muscular weakness sometimes associated with neurodevelopmental disorders. They articulated both the clinical and emotional challenges of this complex monitoring process, at times questioning whether the quality of life of these children treated with DMTs could be considered optimal.

Stopping the progression of the disease does not prevent the child from facing an extremely difficult life with disabilities, whether respiratory, nutritional, etc. (Doctor 2)

Nevertheless, this complexity was not always identified as a PC situation or was differentiated from what specialists referred to as a ‘real PC situation’

A disability that is more or less functional without putting their life at risk. So, at that point, you enter into palliative care in the broad sense, which I provide with physiotherapy, psychomotor therapy, occupational therapy and equipment. (Doctor 11)

So, it’s not what we call true palliative care […] and today we have something that improves the prognosis, but it’s palliative care. (Doctor 15)

### PPC team role in a landscape of therapeutic innovation

As therapeutic innovations continue to emerge, the necessity of a PC approach in SMA type 1 has become increasingly unclear and the role of specialised PC has been redefined by SMA specialists. This theme explores participants’ perspectives on the current role of PPC teams, as understood and defined by the former.

The involvement of a specialised PPC team is only sought if a relationship has already been established with these teams. Three physicians reported never involving a PPC team in the management of children with SMA or in any paediatric neurology cases. They provided PC themselves—out of habit, on the basis of their clinical experience, or due to the time required to involve the PPC team—considering that such support was not necessary for them.

We try to do it ourselves because we are available, and physiotherapists and nurses have this palliative quality. (Doctor 15)

When practitioners have an established connection with the PPC team, DMTs influences the involvement of PPC teams. Indeed, they include them in the care of children with untreated SMA, whereas the question of the place of the PPC team when children are treated seems to be open to debate. Few practitioners introduce them case by case, with or without treatment, but most of them assume that they do not need their involvement as the prognosis as changed.

So, once it has been decided that we are not implementing innovative therapy, well that’s where, at least in my experience, we involve the palliative care team […]. (Doctor 3)

So, it’s true that overall the palliative care teams will be much less called upon for this successive palliative support that will evolve toward end-of-life. (Doctor 11)

I don’t have spinal muscular atrophy, except those without treatment who are in a… we are rather on, truly, life projects. (Doctor 4)

No participants reported involving a PPC team early in the care of children receiving DMTs. Over the course of the interviews, a few practitioners expressed openness to involving PPC teams in the future, but at a later stage, further removed from the initial phase of treatment, particularly in situations of acute decompensation. One physician even suggested introducing the PPC team to families early on as a way to help them cope with the emotional and practical burden. These views were also expressed by some practitioners caring for infants identified through neonatal screening and treated presymptomatically with DMTs, as few situations could still be complex.

So, it’s true that, regarding SMA, I think that, uh… the dual follow-up by the palliative care team could have a real benefit for these severe forms that we don’t support, that we don’t support, that we treat but that remain severe and that we follow over the long term with a heavy disability (Doctor 6)

Only one practitioner mentioned the potential role of the PPC team in the treatment decision-making process, specifically in supporting families. None of the participants suggested that the PPC team could contribute to the decision-making process among specialists, despite many acknowledging that such decisions can be ethically complex and challenging.

But then, well, to possibly help them make the decision. (Doctor 2)

### Key elements driving the involvement of a PPC team

All practitioners who involved PPC teams in the care of children with SMA type 0 and 1 highlighted the different roles that made their contributions valuable. Some tasks seemed specific to PPC teams and required specialised PC expertise, whereas others appeared to be nonspecific but nonetheless helpful—often owing to time constraints or limited capacity within the hospital.

The participants acknowledged PPC teams for their specialised skills, particularly their clinical expertise, their input in treatment-limitation decisions, and the innovative perspectives they brought to medical teams.

They have a real, well, a real added value in these circumstances: considering the situations, considering the deterioration and how to respond to it on the basis of each symptom. All of that is truly… And then they have resources that we don’t necessarily have, such as what we can do in terms of sedation via a continuous or intermittent nasogastric tube, in terms of… Well, in terms of analgesia… So, it’s true that it’s quite helpful. (Doctor 10)

They were also seen as providing essential emotional and professional support to healthcare providers while promoting a broader PC approach.

Finally, it’s extra help to manage all of this. It’s better than having to manage it alone! Because we’re not, we’re not trained…(Doctor 8)

Many practitioners appreciated the flexibility of these teams, as well as their role in coordinating care across hospital settings, community providers, and families. Notably, PPC teams were perceived as the only actor capable of offering continued support to families beyond the child’s death.

So, they adapt to demand. That is, they never… They don’t necessarily put themselves forward if that’s not what the team wants. So, in fact, that’s a big advantage, because… We’re lucky to be able to work with them in a simple way, on the basis of our requests. (Doctor 13)

In fact, I’m thinking, they also manage the aftermath, and that’s good too. So, they follow up with the families afterwards, because ultimately, our work ends there… And I think that’s a good thing, because we are paediatricians for the child, not paediatricians for the family. (Doctor 6)

Some of the skills attributed to PPC teams by participants appeared less specific to PC itself and were instead dependent on local organisational factors. Psychological support for families, for example, was frequently mentioned but could also be provided by the team psychologist if such a position existed.

Well, it’s psychological support too, because there’s a psychologist in the mobile palliative care team. She’s not necessarily available when I see the children for their initial consultation, but she can provide follow-up care. So that’s useful. (Doctor 12)

Several PPC teams offered full-time on-call support, which was particularly valuable when the referring team was unavailable.

Well, first they are present… There’s a doctor and a nurse on call 24 hours a day. So, they’re present, or at least they’re available for families. (Doctor 7)

Home visits were also commonly carried out by PPC teams, including in areas typically inaccessible to referring teams or to standard in-home care services.

But it’s true that we have a palliative care team that travels long distances, even outside the region, to support the families, because otherwise we’re a bit in a desert. (Doctor 14)

Some PPC teams were combined teams that specialised both in pain management and in PPC, and practitioners valued their expertise in this domain. For some, this also provided an opportunity to introduce supportive care to parents of children with SMA type 0 and 1

But no, no, we can call on them for children who are not at the end of their lives, in palliative care in the strict sense of the term, but who are suffering from pain. (Doctor 8)

Six practitioners perceived PPC teams as professional colleagues or integral members of their care network. They primarily referred to them because of their local availability and indicated that they would be less likely to involve them in other circumstances.

I don’t know if we should say “teams”, because I don’t have a palliative care team, I have a doctor and a team with nurses, etc., who work in palliative care in my hospital. So, for me it’s not the palliative care team, it’s [PPC doctor’s name]! Tomorrow, if it’s not [PPC doctor’s name] anymore, if it’s Jules, it might not be the same thing! (Doctor 2)

In common practice and before the widespread involvement of PPC teams, practitioners often had to manage complex situations on their own. Senior physicians frequently recalled the profound sense of isolation experienced by them and by parents, the latter being often left alone at home following the diagnosis.

That is to say that me, before there were mobile palliative care teams, uh well me, I managed everything on my own, you know! (Doctor 12)

Because it is true that when I was an intern, at the beginning of my career, and then as a young senior, we would announce a diagnosis, we had no treatment, people would leave with their baby at home, on their own. And they would carry that burden, you know. (Doctor 12)

At that time, it appeared to be common practice to intubate children with SMA type 1 or perform other invasive procedures without prior collegial discussion. The participants expressed a sense of relief regarding the evolving attitudes and practices surrounding PC situations: collegial discussion and the introduction of specialised PPC in France regarding SMA type 0 and 1 children allowed them to take a step back and reflect on what interventions seemed reasonable.

So, I lived through that era, and I have a clinical case that goes up to age 17, a type 1 who was, let’s say, in a state of extreme disability, meaning arthrodesis, orthoses everywhere, lower limbs, upper limbs, tracheotomy, ventilated. And then at one point there was… It’s dreadful, there, the life of these children, it is no longer possible. (Doctor 5)

### Obstacles and beliefs limiting the involvement of a PPC team

The participants identified several barriers to the introduction of PPC teams—organisational, personal, or semantic—all related to concerns about what place the PPC team would occupy and what parents would find more acceptable and helpful in their opinion.

First, most physicians who involved a PPC team in the care of children with SMA type 0 and 1 emphasised the importance of remaining the patient’s primary coordinator. They considered it essential to remain the child’s primary physician until the very end of care: delegating this role would have felt, to them, like an act of abandonment.

And then all of this mustn’t be diluted among too many people, often there is a referring doctor, and that’s how it should stay. Patients and families need a referring doctor. (Doctor 5)

I am not transferring the patient’s care to palliative care. […] It’s not saying: Well, now we’ve done all we can do. So now you take over for the end, okay? No! (Doctor 2)

Five physicians preferred to rely on other specialists for PC expertise. Paediatric and neonatal intensivists were especially viewed as equally competent in EOL care, decision-making and clinical management in these situations, particularly for type 0 SMA children. Clinical experience was perceived as being at least as important as formal PPC qualifications.

So, they stayed in the neonatal intensive care unit. That’s it. It’s the unit with the most experience in palliative care in neonatology. (Doctor 10)

In the context of home care, the participants expressed differing views regarding the involvement of PPC teams, hospital-at-home services, or both. These perspectives varied according to the local context: hospital-at-home services may either serve as an alternative to PPC teams or collaborate with them. PPC teams could be perceived as less involved when a hospital-at-home service was providing care.

[…] because you never know who does what. And [local PPC team] well, I think they’re handing over to [local hospital-at-home service], so it feels like they’re less present, and that they only come when things are getting truly bad. (Doctor 1)

For four practitioners, the difference between hospital-at-home service and PPC remained unclear, as the former was perceived as being able to implement EOL care plans at home.

When patients aren’t close to the hospital or we can’t because the situation is so difficult, that’s for sure: hospital-at-home, palliative care… There’s a lot of that when they’re often hospitalised and we have to make decisions not to…’(Doctor 15)

Two practitioners distinguished between the two, viewing hospital-at-home services as primarily focused on nursing care, whereas PPC teams were responsible for adapting treatments and overall care plans.

No, because hospital-at-home services will be more about nursing care, but in terms of direct care, um… If I need nutrition, etc… It will be… More like that… That type of activity. Palliative care will be there to ensure, um, comfort… Organisation, the absence of pain, a suitable environment, etc… It’s not quite the same, the same tasks. (Doctor 14)

At times, PPC teams were perceived as excessive and as adding complexity. This could be due to either communication challenges with families or constraints related to limited time. The territorial organisation of services—given that PPC teams operate at a regional level—could also hinder care provision.

So, in fact, I handled this with the coordinating nurse. That meant they knew two people, and I think that was enough, especially since we needed a translator every time, who was always someone different, and that too… It adds to the complexity. In fact, third parties… If the connection is difficult to establish, it’s complicated anyway, so… (Doctor 13)

One practitioner further emphasised the complexity arising from the strict adherence required by the PPC teams to the legal framework.

In addition to the barriers and beliefs previously mentioned, the timing of PPC teams’ involvement also seemed to be an obstacle. Among untreated children, all practitioners agreed on involving PPC teams from the outset when deemed necessary.

So finally, for me, when at the beginning I announce the catastrophic illness and the dire prognosis, if I immediately mention the benefit of having a local team to review the key points and support the child, it goes much more smoothly! (Doctor 1)

In the care of treated children, only a few practitioners indicated that the involvement of a PPC team might be considered later in the trajectory, particularly when pain, discomfort, or respiratory failure emerges. This assumption was based on their experience with other illnesses, given the limited hindsight regarding the trajectories of children treated with DMTs. One practitioner also described arranging a preoperative consultation with the PPC team prior to scoliosis surgery.

Yes, that’s right, and then when you start to have a lot of hospitalisations, a lot of intensive care, a lot of treatment, a lot of surgery plans, and you sometimes ask yourself Is it reasonable to put this child through all this? Shouldn’t we take a step back and say… That… That we should stop? Well, we’re not going to go through with all these projects.… And it’s true that they often intervene in these situations too. It’s not just for types 0 and 1, in fact. (Doctor 8)

The semantics surrounding PC were also perceived as potential obstacles, and the terms employed could differ depending on this belief. In several French regions, PPC teams are multidisciplinary—combining PC and pain management—which creates an opportunity for some practitioners to introduce the concept of PC to parents in a manner that they perceived as more accessible and acceptable. Approximately half of the practitioners introduced the teams under the ‘pain management team’ or ‘supportive care’ role, whereas the other half believed that the term ‘palliative care’ should be explicitly used and thoroughly explained.

We talk about supportive and comfort care, uh, and more, well, focusing on the child’s comfort, and we don’t write the word ‘palliative’ on the foreheads of the people who come through. (Doctor 8)

What’s frightening is saying that the child is going to die! It’s not truly about introducing palliative care. So, from the moment we had a clear discussion with the family that is, ‘‘Well unfortunately there is no solution, he is going to die’’, the palliative care team is actually not a problem! What is complicated is not saying it! (Doctor 13)

Finally, once practitioners considered it appropriate to involve PPC teams in the care of children, they perceived that the teams depended largely on the significance parents attributed to it. Consequently, the level of their involvement varied accordingly.

And then, depending on the wishes of the families, to know to what extent they want to be accompanied by this team. However, in any case, it is systematically offered. (Doctor 3)

## Discussion

Our study contributes to understanding the perceptions of PPC in SMA type 0 and 1 among French paediatric neurologists and paediatric PM&R specialists. We identified 5 themes to highlight the key elements needed to understand their perspective: self-perceived efficacy in PC, general perception of the paediatric palliative approach, the role of the PPC team in a landscape of therapeutic innovation, key elements driving the involvement of a PPC team, and obstacles and beliefs limiting the involvement of a PPC team. These themes will serve as a foundation for analysing the relevance of PC and the benefits of specialised PPC teams in SMA type 0 and 1, as well as the challenges associated with implementing such teams when required.

### Relevance of PC for untreated and treated SMA type 0 and 1 patient

Before the introduction of DMTs, SMA type 0 and 1 was regarded as a life-limiting illness according to the National Hospice and Palliative Care Organisation’s definition [23], and the participants in our study aligned with this point of view in considering all untreated children, all SMA type 0 and untreated SMA type 1, with a clear short term death, a clear indication of PPC.

However, with the advent of DMTs, the boundaries of PC in SMA type 1 have become less clear. The efficacy of these treatments depends on two major factors: their initiation during the presymptomatic phase and the presence of three or more copies of the SMN2 gene in the patient [12, 44–47]. Treated children with SMA type 1 are therefore particularly prone to experience persistent disability and prolonged survival has led to the emergence of new clinical challenges. Motor impairment often persists and varies in severity, with motor abilities ranging from independent walking to independent sitting and to the capacity to maintain motor milestones [12, 46–48]. Some patients require intermittent non-invasive ventilatory support, either during the night or for more than 16 h per day. A small proportion require tracheostomy [12, 48]. There is also a persistent risk of dysphagia, which may lead to the need for nutritional support, for patients with SMA type 1 treated after symptom onset [49]. Pretreated SMA children, particularly those with more than two copies, showed little to no occurrence of these outcomes [47]. The risk of scoliosis has also emerged as a significant concern, particularly in children with SMA type 1, in whom scoliosis is often early and severe [12, 49]. Together, these impairments increase the complexity of care. This clinical fragility may result in repeated hospitalisations and can contribute to premature death.

In our cohort, three physicians did not consider the complexity of care for certain children with treated SMA type 1 to constitute a PC situation. Four described the treatments as semi-curative, and three stated that these were not “pure” PC situations. Only two clearly identified treated children with SMA type 1 as being in a PC situation.

According to C. Agosto et al., multidisciplinary complex care is still needed [21], and in the definition mentioned above, these cases still meet the PPC criteria, as treated SMA type 1 can now be classified as a complex chronic condition [22, 24]. However, DMTs and PC may be perceived as mutually exclusive. For example, in Duchenne muscular dystrophy, American researchers reported in a review being “optimistic in providing a DMT rather than a PC treatment option“[50]. This perception is also reflected in our study. However, the consensus-based definition of PC by the International Association for Hospice and Palliative Care’s members defines a PC situation in relation to severe illnesses as “a condition that carries a high risk of mortality, negatively impacts quality of life and daily function, and/or is burdensome in symptoms, treatments, or caregiver stress” and explicitly includes the possibility of providing specialised PPC in parallel with DMTs [51]. Elverson et al., in a literature review, specifically mentioned SMA as an example of illness where PC ‘is likely to continue to have a role in supporting patients even when “curative” treatment – Nusinersen in the study - is being pursued, where there is significant symptom burden or uncertainty about prognosis’[33]. Five specialists in our study also agreed with the possibility of concurrent care: combining curative treatments and PC care. PPC specialised teams could still play a crucial role in improving care quality, easing caregiver burden, and complementing disease-specific management for children receiving DMTs.

However, the optimal timing for involving specialised PPC teams, as well as the symptoms prompting such involvement, has yet to be determined in treated children with SMA type 1. A recent publication from C. and F. Benedetti also suggested in a postscript letter that PC may not be necessary for children with SMA who initiate DMTs in the asymptomatic or mildly symptomatic phase [52]. While children treated during the asymptomatic phase often fare well, both the literature on DMTs cited before in the introduction [9–19] and our participants describe more uncertainty when symptomatic at treatment initiation and the threshold for defining a PC situation remains ill-defined for SMA specialists. As suggested before DMTs by neurologists, during the International Conference on SMA Standard of Care [29], PC could be an option for nonsitters, as they are symptomatic and more prawn to medical complexity. An alternative approach would be to systematic involve specialised PPC teams immediately upon the diagnosis of SMA type 1, regardless of whether treatment is initiated.

### Expected benefits of specialised PPC teams for SMA type 0 and 1 patient

First, participants noted that the integration of specialised PC in SMA type 0 and 1 management has contributed to a reduction in the use of invasive supportive measures. Interestingly, only a few participants referred to the increase in such interventions following the introduction of DMTs reported in the retrospective data analysis of M. Hully et al. [20]. PPC teams have expertise in this area and may be involved in such cases.

Practitioners also described other specific skills within PPC teams: the support provided to hospital teams and families, specific PC knowledge or having a peculiar vision. These findings were also reported by Firn et al. [53]. In this narrative synthesis and in a state-of-the-art about PC in neonatal neurology [36], spiritual competencies were highlighted as an essential component of specialised PPC, whereas our participants did not mention them. This discrepancy may reflect a different cultural approach in France, as most of the articles originated from Anglo-Saxon countries, and could be an interesting topic for French PPC teams to explore.

Most of the practitioners in our study valued the coordination of care that PPC teams can offer, as described by other neurologists in neonatal neurology [36] and in Duchenne muscular dystrophy [54]. The availability of PPC teams during off-hours and weekends was also highlighted as particularly valuable by several of our participants when such services were available locally, a finding reported as well by Firn et al. [53]. Similarly, an English scoping review about children with life-limiting and life-threatening conditions emphasised the lack of 24/7 access to providers as a barrier to referral. This review also insisted on the desire to reduce caregiver burden as a facilitator in introducing a PPC team [40], which may explain the frequent mention of psychological support. Bereavement support was especially highly appreciated by the participants in our study when accessible, and was likewise emphasised as important by bereaved parents in SMA [55]. However, psychological support appeared to be considered a specific need, primarily for care teams, untreated children, and bereaved families, whereas treated children often already had access to a psychologist within their care team. In an Italian retrospective study, Santini et al. highlighted the essential skills required for PPC psychologists, including flexibility—previously noted by participants regarding PPC teams—as well as transversal skills needed across different care settings [56]. The Italian PPC teams went a step further by developing a dedicated skills curriculum for both the team as a whole and for psychologists specifically [57].

In addition, practitioners highlighted the provision of home care as an important aspect of PPC team involvement, adding continuity and improving family support However, as this approach does not appear to offer clear specificity for practicians compared with hospital-at-home care, it may also act as a barrier to the introduction of specialised PPC teams.

### Implementation challenges of specialised PPC teams in SMA

Several factors limiting the integration of a specialised PPC team were identified. First, PPC is frequently associated with EOL care in qualitative studies exploring the perspectives of paediatric health care professionals [58, 59], a widely held misconception among our participants. Only one practitioner explicitly defined and differentiated the EOL phase from the PC phase, describing the EOL care as being a part of PC closer to the agonic phase. The concept of EOL is not consensual in the literature and is associated with a survival time frame ranging from days to years in a systematic review examining the definition of different terms linked with PC care [60]. The concept of EOL care seemed as personal and subjective as the concept of PPC as, for some, PPC was not always equated with imminent death but was linked to a high certainty of death at a young age or to profound intellectual and multiple disabilities. Importantly, they appeared to distinguish between the complexity associated with PIMD and that of children with SMA type 1 receiving treatment, given the availability of therapeutic options and the uncertainty surrounding long-term outcomes. Evolving uncertainty may challenge clinicians’ perceptions of a PC situation, while DMTs introduce a new dimension of prognostic uncertainty for paediatricians.

Prognostic evolution influenced the palliative approach of French specialists in our study, as only untreated children are consistently identified as needing PPC. This has also been recognised in a recent English scoping review of children with life-limiting and life-threatening conditions [40]. In this 2024 scoping review, neuromuscular diseases were still more likely to be referred to a PPC team, but all the included articles on neuromuscular diseases were published prior to the introduction of DMTs [40]. The uncertainty introduced by DMTs in neuromuscular disease constitutes a novel and complex dimension in this decision-making process [61]. It may reshape both the timing and the perceived necessity of specialised PPC involvement. Some paediatric neurologists and paediatric PM&R specialists in our study considered that the majority of the children they treated met the criteria for a PC situation according to current definitions. However, they did not routinely involve PPC teams in every eligible case. A few participants even reported that they provide PC themselves, stating that they possess the necessary skills, time, and experience. Similar findings have been reported in semi-structured qualitative interviews in oncology, where clinicians also expressed a high level of self-efficacy in addressing PC needs [62]. These views are consistent with Quill et al.’s perspective on the present role of the treating specialist in PC. They suggested initiating a specialised PC consultation only for complex or refractory problems [35]. However, this self-perception contrasts with international standards for PPC which define specialised PPC as being delivered by healthcare professionals “who work exclusively in a PPC setting and should assume the care of families of children with complex needs” [25]. With basic training and experience paediatric neurologists and paediatric PM&R specialists could provide generalised PPC. Indeed, adult neurologists [27] and paediatric neurologists [36, 63] underlined the necessity of having PC training and basic PC skills to improve the quality of care of their patients. In addition, they expressed the need for neurology education for PC specialists. In fact, confidence in their disease-specific expertise may have facilitated or limited referrals to PC teams in our study. This finding was also reported in qualitative interviews in oncology [62] and in the narrative literature of inpatient generalist palliative care providers by Firn et al. [53].

Only a few practitioners reported involving a PPC team before surgery or for chronic pain management in children with treated SMA type 1 or 2. While one specialist highlighted the potential value of early PPC involvement, others preferred a later introduction to avoid confusing parents, who might struggle to reconcile DMTs with the term ‘palliative care’. The difficulty of introducing the terminology of PC is a known barrier among generalist and specialist health care providers and in paediatric specialties because of the presumed negative association between PC and death in the minds of families [62, 64, 65]. This highlights a potential gap in practice: PPC teams are recognised as both valuable and flexible, yet the terminology may still cause hesitation. This issue was also raised in our study by half of the practitioners, but interestingly, the other half emphasised the importance of using appropriate terminology when presenting PPC teams to families, which has not been previously highlighted in the literature, to our knowledge. However, it should be noted that none reported introducing these terms for treated children, and the term “palliative care” may still be perceived by some as a barrier in the context of DMTs, even though the expertise of PPC teams may be recognised as valuable. In a qualitative study among cancer patients, reassuring them that they were not at the EOL while emphasising the potential benefits of PC appeared to facilitate its introduction [66]; this approach could represent a useful strategy to consider.

In addition, for the physicians in our study, maintaining their role as the primary referring physician for the patient was important—an attitude that aligns with concerns expressed by nonspecialist PC practitioners mentioned in the literature review by Firn et al. [53]. However, few participants recognised the capacity of specialised PPC teams to adapt their practices easily to different hospital paediatric teams and families and to enable them to continue fulfilling their role.

### Considerations for implementing a specialised PPC team in SMA

In our study, the progressive expansion of PPC teams in France and the growth of their expertise were acknowledged, which may have facilitated increased referrals. The participants in our study also identified perceiving PPC team members as colleagues as facilitators of their involvement, which seems to be a new observation in the literature.

However, implementing a specialised PPC team still appears challenging, and we propose several avenues for future exploration. First, both SMA specialists and specialised PPC teams could benefit from targeted training from each other to better understand the current challenges faced by children with SMA and the potential role of PPC in this context.

The development of standardised guidelines defining what constitutes a palliative situation and when to involve a specialised PPC team—through consensus among paediatric neurologists, paediatric PM&R specialists, and PPC teams—could also provide a valuable tool to support referrals, as such frameworks have been suggested to enhance referral practices to PC teams for children with life-limiting and life-threatening conditions [40]. Identifying PPC needs in children with SMA can be challenging, and two main approaches emerged: introducing PPC at the time of diagnosis, before the initiation of DMT, or in response to specific emerging needs. We suggest that prognosis should not be the determining factor, given the high level of uncertainty associated with disease trajectories.

Futhermore, as suggested by participants, parents are key to introducing PPC teams, and they are sometimes perceived as barriers to introducing PPC teams from paediatric health care providers [67]. PPC involvement should be guided by parental decisions rather than solely by professionals and could always be offered when a PC situation is identified. In qualitative interviews with paediatric oncologists, positive family feedback reinforced future referrals [62].

Finally, given the improved prognosis of children with SMA type 1 treated with DMTs, a multiprofessional reflection is warranted on whether these children should remain classified by International Standards for PPC as having “progressive conditions without curative treatment options, for which treatment is exclusively palliative and may commonly extend over many years” (Group 3) or be reconsidered under “life-threatening conditions for which curative treatment is possible but may fail for which access to PC services may be necessary due to the complexity of the patients’ needs” (Group 1) [25]. Thus, defining treated SMA type 1 as a “life-limiting condition” may also need to be reconsidered, potentially framing it instead as a “life-threatening condition”. Such a reconsideration could better reflect current clinical reality while acknowledging the ongoing uncertainty and complexity inherent to the condition.

### Strengths & weaknesses

Our study is the first to explore how DMTs influence the PC vision of neurologists and PM&R specialists and the involvement of PPC teams in SMA type 0 and 1. Interviewing sixteen participants on fifty-eight provided a broad overview of the perspectives of paediatric neurologists and paediatric PM&R specialists in SMA in France. The sample was also diverse in terms of age, gender, and geographical location, allowing for discussions from a variety of perspectives. Nearly all participants were based in university hospitals, reflecting the concentration of SMA expertise in tertiary centres in France, which may have influenced perspectives on PC and its accessibility. Most patients are primarily managed by paediatric neurologists, as seen in our cohort and consistent with clinical practice, though paediatric neurologists and PM&R specialists have distinct training and objectives that may shape their views. Participants varied in age but showed less gender diversity, with no clear opinion differences by gender. The only noticeable pattern was related to age: younger participants appeared more familiar with the current conceptualisation of PC and reported involving paediatric palliative care teams earlier and in a wider range of situations.

Several limitations should be noted. First, in the demographic questions regarding patients, we only collected the number of new patients followed over the past year. Given the low incidence of SMA, a single year may not provide a fully representative picture, which explains the absence of new patients for some physicians. Nevertheless, the reported numbers appeared consistent and representative when compared with the number of new patients included over three years in the study by Desguerre et al. [12]. A longer timeframe could have provided additional insight but was avoided to minimise recall bias. Data on SMA type distribution and on children follow-up by specialised PPC teams would also have been informative.

Second, all the interviews were conducted by a single researcher in the final year of medical school, which may have introduced a hierarchical or rank-related effect during the interviews. In addition, the interviewer was personally known to one participant and was trained both in PC and in paediatric neurology, which may have influenced her perspective during the study. It is acknowledged that a researcher’s opinions and professional status can influence the data collection process. However, since participants held higher-ranking positions than did the interviewer, this may have facilitated more open expression. To enhance reflexivity, a diary was maintained, group reflections with researchers and medical teams were conducted, and interviews were triangulated.

Third, participants’ responses may have been influenced by social desirability bias, given that PC is both a prominent and sensitive topic, and participants were aware that it was the focus of the study. The anonymization of the transcripts and the assurance of confidentiality may have reduced this bias.

Fourth, participants were not provided with predefined definitions of PPC, specialised PPC, or life-limiting conditions. This approach may have contributed to multiple misconceptions, but it allowed physicians to express their own understanding and interpretation of PPC, and to describe how this perspective influences the integration of a specialised PPC team in the context of SMA and DMTs.

Finally, the resources of local PPC teams may vary depending on the context, which could have impacted participants’ views. Some teams, for example, have only part-time resources, and team composition can differ significantly across settings. This could be viewed as a strength, as the purpose of this study was to include diverse French contexts to explore our research question.

## Conclusion

This study has demonstrated the widespread recognition of the PC dimension in SMA type 0 and 1 in France. With the emergence of DMTs, the definition of a PC situation has become less clear. Untreated children, including all type 0 and a subset of type 1 SMA children, were still generally considered appropriate candidates for the involvement of a PPC team. However, for SMA type 1 treated children, participants mostly reported not involving a PPC team, even in cases of medical complexity, despite a broad definition of PPC that could encompass these children. Nonetheless, a few participants acknowledged the potential positive impact of introducing PPC in such situations. The question arises as to why these situations are not recognised by paediatric neurologists and paediatric PM&R specialists and how the health system can be changed to allow for an earlier introduction.

It would also be valuable to examine the perceptions and opinions of families of treated children with SMA type 1, and to investigate whether the introduction of a PPC team could be offered as a family-centered choice following a systematic presentation.

## Supplementary Information

Below is the link to the electronic supplementary material.

Supplementary Material 1

Supplementary Material 2

Supplementary Material 3

## Data Availability

The datasets generated and/or analysed during the current study are available upon reasonable request from the secure database of the centre, subject to appropriate approvals.

## Abbreviations

2SPP: French Paediatric Care Association
CLB: Léon Bérard Centre
CNIL: French Data Protection Authority
CRCL: Research Cancerology Centre in Lyon
DMTs: Disease-modifying therapies
EOL: End-of-life
NHPCO: National Hospice and Palliative Care Organisation
PC: Palliative care
PM&R: Physical medicine and rehabilitation
PPC: Paediatric palliative care
SMA: Spinal muscular atrophy
UCBL: Claude Bernard Lyon 1 University
