# Supplementary material for: Paediatric palliative approach in the era of disease modifying therapies in spinal muscular atrophy — a comprehensive landscape of French specialist physicians’ perspectives
Source: Orphanet J Rare Dis. 2026 Aug 5;21:392. doi: 10.1186/s13023-026-04481-5 (PMC13733110; doi:10.1186/s13023-026-04481-5)
Supplement: Supplementary file 1 [file 13023_2026_4481_MOESM1_ESM.docx]

**Appendix 1 – Interview guide**

Hello Dr [last name], thank you for agreeing to participate in this study on type 0 and 1 infantile spinal muscular atrophy and paediatric palliative care.

My name is Marie-Liesse Guenée, and I am currently in my research year as part of a master's degree in palliative care at the Léon Bérard Centre Human and Social Sciences (HSS) laboratory, working more specifically with the ESPPéRA research team.

If you agree, we will discuss your experience with the evolution of care for type 0 and 1 SMA. This interview will be recorded and transcribed. It will be pseudonymized, and the recording will be deleted after transcription. This interview will last approximately 45 minutes to 1 hour. You may stop the interview at any time if you wish.

Do you have any questions before we begin?

First, could you tell me about your experience with SMA?

| Theme | Question | Follow-up question | Purpose of the question |
| --- | --- | --- | --- |
| Emergence of a new treatment and paradigm shift | In what ways has the introduction of treatments for types 0 and 1 SMA impacted your day-to-day practice? | Could you describe how a child's trajectory has changed from the beginning of your career until now? For children with type 0? Type 1? | Perception of current and past disease trajectories |
|  | In what ways have new treatments changed the lives of children with types 0 and 1 SMA? | What are the differences in terms of symptoms since these treatments became available? | Perception of paradigm shift |
| New questions have arisen from the emergence of treatments | What are the current needs in terms of supportive care for treated and untreated children? | What complementary care is currently being provided?  Has this changed since treatments became available? | Exploration of the supportive care provided alongside treatments |
|  | Since these treatments became available, have you identified any new issues for patients, their families, and healthcare teams? | Are there any differences in terms of follow-up care, the equipment used, or the potential for complications? | Exploration of expected and unexpected adverse effects and disabilities |
| Evolution of the decision-making process | Which factors do you consider when deciding on a course of treatment for children with types 0 and 1 SMA? | Which factors influence the decision to treat, or not to treat, children with SMA? | Exploring treatment indications |
|  | What changes have you noticed in the criteria for access or nonaccess to treatment? | Have you noticed any changes in treatment decisions, based on your current perspective and the knowledge you have gained from patients who have already been treated? | Impact of monitoring treated patients on current treatment decision-making |
| The role of PPC teams in practice | How would you describe your current working relationship with the PPC resource team in your region? | Do you work alongside this team on a daily basis? | Habit of integrating a PPC team |
|  | What do you think the role of these teams is in SMA? | How do you perceive the role of the paediatric palliative care team in SMA? | Perception of PPC teams' missions in SMA |
| Current integration of a PPC team | When do you think it is most suitable to initiate a discussion about integrating a PPC team with the child and family? | What factors would lead you to initiate a discussion about integrating a PPC team with the child’s family? | Exploring the timing of PPC team involvement with children and families |
|  | How do you introduce a PPC team to families and children? | What terms do you use to present a PPC team to families and children? | Investigating the ways physicians implement a PPC team |
|  | Since DMTs have become available, what is the role of PPC teams in types 0 and 1 SMA? | How have you integrated them since the introduction of new treatments? | The role of PPC teams since the introduction of DMTs |
|  | What do you expect from PPC teams in the future of SMA, particularly with the introduction of neonatal screening? | What role would you assign to PPC teams in the future of SMA? Do you think this role will change with early screening? | Exploring the perceived role of PPC teams in SMA in the future |

Before we conclude, I will ask you a few questions of a demographic nature and questions describing your current active follow-up of children with spinal muscular atrophy.

**“Sociodemographic” questions at the end of the interview**

1. Gender (male/female)
2. What is your age?
   A. < 40 years
   b. 40–49 years
   c. 50–59 years
   d. > 60 years
3. What is your specialty? (Paediatric neurology, PM&R speciality, etc.)
4. In which type of institution do you work? Coordinating reference centre/constituent reference centre/competence centre
5. For how long have you been following patients with neuromuscular diseases (in years)?
   A. 0–5 years
   b. 5–10 years
   c. 10–15 years
   d. 15–20 years
   e. > 20 years
6. Over the past 12 months, how many newly diagnosed children with SMA have you seen?
7. Over the past 12 months, how many of these have been treated with DMTs?
8. Over the past 12 months, how many have you followed who were **not** treated with these new therapies?
9. In the past 12 months, have you had any deaths related to type 0 or type 1 SMA among the children you follow?
10. Are you in a pilot region for neonatal screening?

Is there anything you would like to add? Would you like to discuss a specific topic before we finish? Do you have any questions or comments (about the project, the interview, etc.)?

Thank you, Dr. [last name], for your participation. I would be happy to share the results of this study with you, should you be interested.
