# Supplementary material for: Paediatric palliative approach in the era of disease modifying therapies in spinal muscular atrophy — a comprehensive landscape of French specialist physicians’ perspectives
Source: Orphanet J Rare Dis. 2026 Aug 5;21:392. doi: 10.1186/s13023-026-04481-5 (PMC13733110; doi:10.1186/s13023-026-04481-5)
Supplement: Supplementary file 2 [file 13023_2026_4481_MOESM2_ESM.docx]

**Appendix 2 – Thematic map**
