# Supplementary material for: Paediatric palliative approach in the era of disease modifying therapies in spinal muscular atrophy — a comprehensive landscape of French specialist physicians’ perspectives
Source: Orphanet J Rare Dis. 2026 Aug 5;21:392. doi: 10.1186/s13023-026-04481-5 (PMC13733110; doi:10.1186/s13023-026-04481-5)
Supplement: Supplementary file 3 [file 13023_2026_4481_MOESM3_ESM.docx]

**Appendix 3 – Descriptive characteristics of the SMA specialist participants**

|  | N = 16 | % |
| --- | --- | --- |
| Sexe  Female  Male | 11  5 | 69  31 |
| Age (years old)  < 40  40-49  50-59  > 60 | 5  4  3  4 | 31  25  19  25 |
| Medical specialisation  Paediatric neurologists  Paediatric PM&R | 14  2 | 87  13 |
| Practice setting  Coordinating reference centre for SMA  Constituent reference centre for SMA  Expertise centre for SMA  Other setting | 5  5  5  1 | 31  31  31  7 |
| Regional neonatal screening  Yes  No | 5  11 | 31  69 |

SMA=Spinal Muscular Atrophy

PM&R=physical medicine and rehabilitation

**Appendix 4 – Number of newly followed patients and their trajectory in the year before the interviews of the 16 French physicians specialised in SMA**

|  | On 16 physicians | | Per physician |
| --- | --- | --- | --- |
|  | n | % | mean [min-max] |
| New patients | 52 | 100 | 3.3 [0-7] |
| Newly followed patients in 2024 and their trajectory  Treated *  Type 0  Type 1  Type 2  Type 3  Unknown type  Untreated*  Type 0  Type 1  Type 2  Other**  Unknown type | 34  0  18  6  4  6  18  4  11  1  2  0 | 65  35 | 2.1 [0-5]  1.1 [0-3] |
| Patients deceased in 2024*** | 13 | 25 | 0.8 [0-3] |
|  | On 52 new patients followed by the 16 physicians | | Per physician in a screening program  (n=5) |
|  | n | % | mean [min-max] |
| Patients detected by neonatal screening | 11 | 21 | 2.2 [0-4] |

*** Treatment refers to disease-modifying treatments (Nursinersen, etc)**

**** Diagnosed through newborn screening and presenting three copies of the SMN gene.**

******* One treated patient with type 1 SMA died. For untreated patients: 2 type 0 et 6 type 1 were alive at the time of the interview.
